# Sex- and Age-Related Differences in the Contribution of Ultrasound-Measured Visceral and Subcutaneous Abdominal Fat to Fatty Liver Index in Overweight and Obese Caucasian Adults

**DOI:** 10.3390/nu11123008

**Published:** 2019-12-09

**Authors:** Alessandro Leone, Alberto Battezzati, Giorgio Bedogni, Laila Vignati, Angelo Vanzulli, Ramona De Amicis, Andrea Foppiani, Simona Bertoli

**Affiliations:** 1 Department of Food, International Center for the Assessment of Nutritional Status (ICANS), Environmental and Nutritional Sciences (DeFENS), University of Milan, Via Sandro Botticelli 21, 20133 Milan, Italy; alessandro.leone1@unimi.it (A.L.); lailavignati@libero.it (L.V.); ramona.deamicis@unimi.it (R.D.A.); andrea.foppiani@unimi.it (A.F.); simona.bertoli@unimi.it (S.B.); 2 Clinical Epidemiology Unit, Liver Research Canter, 34149 Basovizza, Trieste, Italy; giorgio.bedogni@gmail.com; 3 Department of Oncology and Hemato-Oncology, University of Milan, 20122 Milan, Italy; angelo.vanzulli@unimi.it; 4 Istituto Auxologico Italiano, IRCCS, Lab of Nutrition and Obesity Research, 20145 Milan, Italy

**Keywords:** fatty liver disease, fatty liver index, visceral adipose tissue, subcutaneous adipose tissue, obesity, ultrasonography

## Abstract

Differences in body fat distribution may be a reason for the sex-, age-, and ethnicity-related differences in the prevalence of fatty liver disease (FL). This study aimed to evaluate the sex- and age-related differences in the contribution of visceral (VAT) and subcutaneous (SAT) abdominal fat, measured by ultrasound, to fatty liver index (FLI) in a large sample of overweight and obese Caucasian adults, and to identify the VAT and SAT cut-off values predictive of high FL risk. A cross-sectional study on 8103 subjects was conducted. Anthropometrical measurements were taken and biochemical parameters measured. VAT and SAT were measured by ultrasonography. FLI was higher in men and increased with increasing age, VAT, and SAT. The sex*VAT, age*VAT, sex*SAT, and age*SAT interactions negatively contributed to FLI, indicating a lower VAT and SAT contribution to FLI in men and in the elderly for every 1 cm of increment. Because of this, sex- and age-specific cut-off values for VAT and SAT were estimated. In conclusion, abdominal adipose tissue depots are associated with FLI, but their contribution is sex- and age-dependent. Sex- and age-specific cut-off values of ultrasound-measured VAT and SAT are suggested, but they need to be validated in external populations.

## 1. Introduction

Fatty liver disease (FL) is the most common chronic liver disease in Western countries [1], and is characterized by an excessive accumulation of fat in the liver. It is usually divided into alcoholic fatty liver disease (AFLD) and non-alcoholic fatty liver disease (NAFLD) [2]. The diagnosis of FL is made through imaging procedures or liver histology. Moreover, Bedogni et al. established a formula to calculate the fatty liver index (FLI) based on routine clinical measurements like body mass index (BMI), waist circumference (WC), triglycerides (TG), and gamma-glutamyltrasnferase (GGT) to predict ultrasonographic FL [3]. This simple, not expensive, non-invasive, and cross-validated [4,5] algorithm has excellent discriminative ability to identify subjects at higher risk of FL.

In the Italian general population, the prevalence of FL is 45–46% [1,3,6]. However, sex-, age-, and ethnicity-related FL prevalence have been reported [7,8,9,10,11]. Many studies have reported that the male sex has a higher risk for FL, with a doubled prevalence compared to women [10,12]. Furthermore, a number of studies have shown that the prevalence of FL increases with age, and that Hispanic individuals, compared to non-Hispanic whites, have a higher prevalence of FL, and non-Hispanic blacks a lower prevalence [13]. Sex-, age-, and ethnicity-related differences in body fat distribution may be one reason for the differences in FL prevalence observed in these different subject groups.

Obesity, both general and visceral obesity, is most probably the main risk factor for FL [13]. However, it is well known that there are sex-, age-, and ethnicity-related differences in the distribution of adipose tissue, particularly in the abdominal compartments. For instance, at the same degree of obesity, women have 10% higher body fat compared to men. Moreover, at the same level of total adiposity, estimated either by BMI or by imaging techniques, women have more abdominal and gluteal–femoral subcutaneous adipose tissue (SAT) than men, who show higher visceral adipose tissue (VAT). Aging, for its part, increases adiposity in both sexes. Indeed, aging is associated with increased VAT accumulation [14]. This increase is greater in women, almost quadrupling between the ages of 25 and 65 years, whereas in men, it is only slightly more than doubled [14]. In contrast, SAT, despite an initial increase with increasing age, starts to decline after 50 years in men and after 60 years in women [15]. Finally, white men store more fat as VAT than black men, while no such difference has been reported for women. However, SAT has been found to be higher in black women than in white, a difference not reported for men [16]. Nevertheless, there have been few studies investigating sex- and age-related differences in the contribution of abdominal fat compartments to FL.

Imaging techniques like computed tomography and magnetic resonance imaging are the reference methods used for the assessment of body fat compartments [15]. However, because they are expensive and because computed tomography exposes the patient to ionizing radiation, imaging techniques are not often applied in routine clinical practice and large epidemiological studies. Given its low cost and simplicity, waist circumference (WC) is the most clinically used surrogate measure of abdominal fat. However, it does not allow separate assessment of VAT and SAT contributions. Ultrasonography (US), however, offers a low-cost, reproducible, reliable, and non-invasive alternative to the gold standard imaging techniques [15].

In the present study, we evaluated, in a large sample of overweight and obese Caucasian adults, the contribution of sex- and age-related differences in ultrasound-measured VAT and SAT to FL. We also identified sex- and age-specific VAT and SAT cut-off values for the identification of subjects at high FL risk.

## 2. Materials and Methods

### 2.1. Study Design and Procedures

We carried out a cross-sectional study on 8316 consecutive overweight and obese Caucasian adults who voluntary referred to the International Center for the Assessment of Nutritional Status (University of Milan, Milan, Italy) between September 2010 and February 2019 for participation in a structured weight loss program.

Each participant was subjected to a clinical examination, an anthropometric assessment, blood tests, and an ultrasound measurement of VAT and SAT, all on the same day. Their smoking status and physical activity level were investigated through a structured interview. Subjects who spent ≥2 h/week in any structured physical activity were considered physically active [17]. Excluded from the study were participants younger than 18 years; those with diagnosed infective, cardiac, neurological, gastrointestinal, pulmonary, or renal disease; those with previous diagnoses of hepatitis B and C; those using medications known to cause lipodystrophy (e.g., steroids and antiretroviral agents); and those who had been subjected to surgery in the abdominal area. From the 8316 participants initially recruited, we excluded those with missing values on one of the variables of interest (142) and those with VAT and SAT higher than 14 cm, because of the difficulty of measurement (71). A total 8103 subjects were included in the final dataset. The study was conducted according to the guidelines laid down in the Declaration of Helsinki, and all procedures involving human participants were approved by the ethics committee of the University of Milan (report n. 23/2016). Written informed consent was obtained from all participants.

### 2.2. Clinical and Anthropometrical Examination

Detailed medical interviews were conducted to investigate the clinical history of the patients and their current drug therapies. Anthropometric measurements were taken following international guidelines [18]. Body weight was measured to the nearest 100 g with a column scale (Seca 700 balance, Seca Corporation) and with participants wearing only light underwear. Body height was measured to the nearest 0.1 cm using a vertical stadiometer. BMI was calculated as weight (kg)/height (m)^2^ and classified as follows: overweight = BMI 25.0–29.9, obese = BMI ≥ 30. WC was measured at the midpoint between the last rib and the iliac crest with a non-stretch tape to the nearest 0.5 cm.

### 2.3. Abdominal Ultrasonography

Abdominal ultrasonography was carried out on the fasting patients by the same physician, using a Logiq 3 Pro instrument equipped with a 7.5 MHz linear probe and with a 3.5 MHz convex-array probe (GE Healthcare). VAT and SAT were measured 1 cm above the umbilicus. The measurements were taken at the end of expiration and applying a standardized probe pressure. SAT, measured with the 7.5 MHz linear probe, was defined as the distance between the epidermis and the external face of the rectus abdominis muscle; VAT, measured with the 3.5 MHz convex-array probe, was defined as the distance between the anterior wall of the aorta and the posterior surface of the rectus abdominis muscle [19,20]. Each measurement was taken three times and the mean of the three measurements was entered into the database.

### 2.4. Blood Sampling and Fatty Liver Index Determination

Fasting blood samples were obtained between 8:30 and 9:00 AM and immediately analyzed at the ICANS laboratory. Triglycerides and GGT were measured by means of an enzymatic method (Cobas Integra 400 Plus, Roche Diagnostics, Rotkreuz, Switzerland).

Fatty liver index was calculated using the following formula [3]:

FLI = (e^0.953 × loge (triglycerides) + 0.139 × BMI + 0.718 × loge (ggt) + 0.053×waist circumference − 15.745^)/(1 + e^0.953 × loge (triglycerides) + 0.139 × BMI + 0.718 × loge (ggt) + 0.053 × waist circumference − 15.745^) × 100

FLI varies between 0 and 100, and a FLI ≥ 60 has been associated with a high probability of FL [3].

### 2.5. Statistical Analysis

Many continuous variables followed a non-Gaussian distribution and all are reported herein as 25th, 50th, and 75th percentiles. Discrete variables are reported as numbers and frequencies. In order to limit the influence of outliers, we winsorized, using a tail of 0.01, all continuous variables besides age. This means that values under the 1st or over the 99th internal percentile were set as equal to the 1st or 99th internal percentile, respectively. This limitation of the influence of outliers is an important strategy to increase the generalizability of regression models [20]. Linear regression models were used to assess the association of VAT and SAT with FLI. Included in the models as predictors were sex (discrete, 0 = women, 1 = men), age (continuous), smoking (discrete; 0 = never smoked, 1 = former smoker, 2 = smoker), and structured physical activity (discrete; 0 = no, 1 = yes). Sex and age interactions with SAT and VAT were also included in the model. We used multivariable fractional polynomials to model the non-linear associations of continuous predictors with the outcome. Using this approach, we found that a square root transformation of VAT/10 (VAT/10)^0.5^ and a squared transformation of SAT (SAT^2^) ensured better fits for the model. A receiver operating characteristic (ROC) curve analysis was used to develop a cut-off for VAT and SAT associated with the presence of high probability of FL (FLI ≥ 60). We performed exploratory subgroup analysis to obtain the sex- and age-specific cut-off values. We evaluated the cut-off indexes for VAT and SAT that maximized the Youden index (sensitivity + specificity−1). A *p*-value < 0.05 was considered statistically significant. Statistical analysis was performed using STATA version 12.0 (StataCorp, College Station, TX, USA).

## 3. Results

The general characteristics of the study population are reported in Table 1

In our population, 51.2% of subjects were at high risk for FL (FLI ≥ 60). The FL frequency was significantly higher in men (OR = 13.9, 95%CI: 11.8, 16.4, *p* < 0.001) and increased with increasing age (OR = 1.04, 95%CI: 1.04, 1.05, *p* < 0.001 per year) and BMI (OR = 2.0, 95%CI: 2.0, 2.1, *p* < 0.001 per kg/m^2^).

Table 2 reports the contribution of VAT and SAT to FLI, taking into account sex, age, lifestyle indexes and their interactions with abdominal fat depots.

Being male and habitual smoking increased the value of FLI, while being physically active decreased it. Moreover, FLI increased with increasing age, VAT, and SAT. The sex*VAT, age*VAT, sex*SAT, and age*SAT interactions negatively contributed to FLI, indicating a lower VAT and SAT contribution to FLI in men and in the elderly for every 1 cm of increment.

Given the sex and age differences in the contribution of VAT and SAT to FLI, we estimated the sex- and age-specific cut-off values for VAT and SAT by dividing subjects into different categories based on birth sex and age decade (Table 3).

VAT cut-offs were higher in men and increased with increasing age in both sexes. In contrast, SAT cut-offs were similar between the sexes and decreased with increasing age. Moreover, in postmenopausal women, SAT optimal cut-offs suffered low specificity and AUC. In men aged ≥40 years, SAT optimal cut-offs, instead, suffered low sensitivity and AUC.

## 4. Discussion

In this study, we investigated the sex- and age-related contributions of abdominal fat depots to FLI in a large sample of overweight and obese Caucasian subjects, and identified sex- and age-specific cut-off values for high probability of FL. Overall, both VAT and SAT were associated with FLI, in agreement with previous studies reporting a relationship between abdominal fat deposits and risk of FL [21]; however, the contributions differed between sexes and across ages.

Obesity, especially abdominal obesity, plays a pivotal role in the development of FL [22]. Excess of abdominal fat leads to an enhanced lipolysis and increased flux of free fatty acids (FFA) towards the liver through portal circulation [22]. Increased hepatic FFA induces increased hepatic lipogenesis and gluconeogenesis [23], as well as decreased insulin clearance, resulting in hyperinsulinemia and insulin resistance [23,24]. Insulin, in turn, promotes de novo lipogenesis [25], contributing to the synthesis of hepatic triglycerides and the promotion of FL [26]. Moreover, the excess of abdominal fat alters the expression and secretion of inflammatory cytokines, like tumor necrosis factor (TNF)-a, interleukin (IL)-6, and IL-8, and of some adipokines, like leptin and adiponectin, also involved in the pathogenesis of FL [21,22]. Indeed, inflammatory cytokines contribute to the activation of Kupffer cells and are responsible for the transformation and perpetuation of hepatic stellate cells to the myofibroblastic phenotype [27,28]. Adiponectin and leptin act respectively, regulating whole-body glucose homeostasis and hepatic insulin sensitivity [29] and promoting fibrogenesis in stellate cells by stimulating the production of fibrogenic genes and inflammation in T cells [30,31]. Evidence shows that VAT is characterized by a higher rate of lipolysis than SAT [32], and thus it contributes more to excessive fatty acid deposition in the liver, with subsequently increased hepatic glucose production, hyperinsulinemia, and hypertriglyceridemia [33]. Additionally, the expression of inflammatory cytokines is increased in VAT compared to SAT [34]. Finally, VAT is more involved than SAT in increasing leptin production and reducing the circulating levels of adiponectin. In the light of this, it is easily understandable that VAT contributes more to hepatic lipid accumulation than SAT [35].

Body fat distribution significantly differs between men and women [36,37,38]. Women have more SAT, whereas men have more abdominal VAT [16,39,40,41]. Moreover, women also tend to store energy in the gluteal–femoral adipose tissue, which is thought to be protective against the adverse effects of obesity [42,43]. This different pattern of body fat distribution may place men at greater risk of FL. In agreement with this hypothesis, our results showed that male sex contributes to FLI. Interestingly, we found that for each unit of adipose tissue, VAT and SAT contributed more in women than in men. This result was also confirmed by the lower VAT cut-off values necessary for women to be at high FL risk. This finding may have multiple reasons. Despite the fact that women generally store fat both at abdominal and gluteal–femoral levels, about 40% of those aged 30–79 years store it predominantly at the abdominal level [37,44], suffering from the same metabolic complication as men [45]. It is possible, therefore, that in women with an upper-body obesity phenotype, abdominal fat depots contribute more to FLI. A further reason could be related to the differences in alcohol intake between the sexes. Chronic alcohol consumption causes the secretion of pro-inflammatory cytokines and reactive oxygen species, which cause inflammation, apoptosis, and finally fibrosis of the liver cells [46]. Moreover, alcohol abuse increases the risk of obesity and triglycerides and GGT levels [47,48], factors used to calculate FLI, even if in the general population of Northern Italy no association was recently found between FL and alcohol intake [6,49]. Compared to men, women are more likely to abstain from drinking alcoholic beverages, drink less, and are less likely to engage in drinking problems [50]. Therefore, alcohol intake may have a greater contribution in men compared to women in explaining FLI variability, reducing that of a single unit of abdominal adipose tissue.

We found that FLI increased with increasing age, suggesting that the elderly have a higher risk of developing FL, in agreement with previous investigations. However, the contribution of a single unit of both abdominal fat depots to FLI diminished with increasing age. To confirm this, the predictive VAT cut-offs of a FLI ≥ 60 increased with increasing age decade. Furthermore, in men aged 40 years or more and in postmenopausal women, SAT had little or no predictive ability for a FLI ≥ 60, as demonstrated by low AUC values and/or low sensitivity and specificity values of SAT cut-off. Aging is characterized by an increase and redistribution of body fat in both sexes [51]. In particular, aging is accompanied by a reduction of abdominal SAT and an increase in abdominal VAT [52]. Lower energy storage in SAT could be the reason for its reduced contribution to FL in the elderly. In contrast, despite VAT being notoriously associated with negative health outcomes, the clinical implications of an age-related shift in VAT remain unclear [52]. Moreover, intra-muscular fat is also increased in aging [52,53]. These fat deposits are commonly associated with metabolic derangements caused by lipotoxicity at the level of the myocyte, which are thought to be implicated in the decline of peripheral glucose tolerance [53,54], increasing the risk of FL [33]. This would lead to a reduction in the unitary contribution from VAT alone.

This was the first study to report sex- and age-related differences in the contribution of ultrasound-measured VAT and SAT to FLI, as well as the first to identify sex- and age-specific cut-off values of both abdominal fat depots predictive of high risk for FL. This information has been obtained in a large sample of Caucasian overweight and obese subjects, allowing a better estimation of contributions and predictive cut-offs. All ultrasound measurements of abdominal fat depots were taken by the same physician, who has had long-term experience in using ultrasonography, thus avoiding inter-operator measurement errors. Moreover, we recruited only Caucasian subjects, avoiding the influence of ethnicity on both abdominal fat distribution and FL risk. On the other hand, this limits the application of these findings to other ethnic groups. Another limitation is that we assessed abdominal fat with ultrasonography, not with a gold standard imaging technique. However, evidence has shown a good correlation between ultrasound measurements of abdominal fat thicknesses and areas by CT or MRI [19]. Moreover, we used FLI to identify subjects at high FL risk, not ultrasonography to diagnose FL. Nevertheless, FLI has been found to be able to predict FL in several populations [3,4,5]. Finally, we did not take into account the patients’ dietary habits, a factor known to be associated with both abdominal adipose tissue [55] and FL risk [56], and which can be different between men and women and change with age [57]. Therefore, future studies might address the ability of diet to mitigate the effect of abdominal adipose tissue on FL.

## 5. Conclusions

In conclusion, abdominal adipose tissue depots are associated with FLI, but their contribution is sex- and age-dependent. Sex- and age-specific cut-off values of ultrasound-measured VAT and SAT could be useful both clinically and in research field to screen overweight and obese populations to identify subjects at risk for fatty liver disease. However, our findings need further validation against a fatty liver diagnosis made through imaging techniques and replicated in external populations.

## Figures and Tables

**Table 1:** General characteristics of the study population.

	Women			Men			Total		
	*n* = 5530			*n* = 2573			*n* = 8103		
	P25	P50	P75	P25	P50	P75	P25	P50	P75
Age (years)	39	48	57	40	48	57	39	48	57
BMI (kg/m^2^)	27.3	29.8	33.6	28.2	30.7	33.7	27.5	30.1	33.7
Waist circumference (cm)	90.3	97.0	105.4	101.0	107.8	116.2	92.9	100.6	109.7
SAT (cm)	2.3	3.0	3.8	2.0	2.7	3.5	2.2	2.9	3.8
VAT (cm)	3.5	4.9	6.7	5.9	7.7	9.6	4.0	5.7	7.9
Triglycerides (mg/dL)	67	90	125	87	122	174	72	99	140
GGT (U/L)	12	17	24	22	31	46	14	20	32
Fatty Liver Index	26.8	49.6	77.0	60.3	80.5	92.3	33.9	61.2	84.9
	***n***	**%**		***n***	**%**		***n***	**%**	
Age classes									
18–19 years	73	1.3		22	0.9		95	1.2	
20–29 years	481	8.7		156	6.1		637	7.9	
30–39 years	941	17.0		461	17.9		1402	17.3	
40–49 years	1525	27.6		771	30.0		2296	28.3	
50–59 years	1383	25.0		627	24.4		2010	24.8	
60–69 years	800	14.5		402	15.6		1202	14.8	
≥70 years	327	5.9		134	5.2		461	5.7	
Total	5530	100.0		2573	100.0		8103	100.0	
BMI classes									
Overweight	2845	51.4		1094	42.5		3939	48.6	
Obese	2685	48.6		1479	57.5		4164	51.4	
Total	5530	100.0		2573	100.0		8103	100.0	
FLI classes									
0–9.9	163	2.9		3	0.1		166	2.0	
10–19.9	709	12.8		37	1.4		746	9.2	
20–29.9	737	13.3		82	3.2		819	10.1	
30–39.9	625	11.3		118	4.6		743	9.2	
40–49.9	562	10.2		180	7.0		742	9.2	
50–59.9	523	9.5		217	8.4		740	9.1	
60–69.9	500	9.0		265	10.3		765	9.4	
70–79.9	489	8.8		365	14.2		854	10.5	
80–89.9	556	10.1		513	19.9		1069	13.2	
90–100	666	12.0		793	30.8		1459	18.0	
Total	5530	100.0		2573	100.0		8103	100.0	
Risk of fatty liver disease									
<60	3320	60.0		637	24.8		3957	48.8	
≥60	2210	40.0		1936	75.2		4146	51.2	
Total	5530	100.0		2573	100.0		8103	100.0	

**Table 2:** Association of visceral and subcutaneous abdominal fat with fatty liver index.

	Fatty Liver Index	Fatty Liver Index
Sex (male)	26.95 ***	29.27 ***
	[22.21,31.69]	[27.67,30.87]
Age (years)	0.41 ***	0.85 ***
	[0.27,0.55]	[0.79,0.91]
Smoking		
Ex-smoker	0.45	1.05
	[−0.58,1.48]	[−0.16,2.27]
Smoker	1.21 *	2.98 ***
	[0.17,2.25]	[1.76,4.20]
Physical activity (yes)	−3.63 ***	−7.12 ***
	[−4.51,−2.75]	[−8.12,−6.13]
(VAT/10)^0.5^	144.18 ***	−
	[135.42,152.93]	
Sex*(VAT/10)^0.5^	−25.80 ***	−
	[−31.07,−20.53]	
Age*(VAT/10)^0.5^	−0.58 ***	−
	[−0.75,−0.41]	
SAT^2^	−	2.32 ***
		[2.10,2.53]
Sex*SAT^2^	−	−0.46 ***
		[−0.57,−0.35]
Age*SAT^2^	−	−0.02 ***
		[−0.02,−0.01]
Intercept	−49.29 ***	−4.4 **
	[−56.17,−42.40]	[−7.48,−1.33]
Observations	8103	8103

Values are linear regression coefficients and 95% confidence interval (in brackets). Abbreviations: * *p* < 0.05, ** *p* < 0.01, *** *p* < 0.001.

**Table 3:** Sex- and age-specific cut-off values of VAT and SAT predicting a high risk of fatty liver.

Sex	Age (Years)	*n*	Optimal Cutoff									
			VAT					SAT				
			VAT	(VAT/10)^0.5^	SN	1-SP	AUC	SAT	SAT^2^	SN	1-SP	AUC
Women	18–29	554	4.40	0.66	0.65	0.14	0.812	3.86	14.90	0.82	0.27	0.857
	30–39	941	4.79	0.69	0.69	0.20	0.811	3.41	11.63	0.76	0.34	0.775
	40–49	1525	4.88	0.70	0.81	0.26	0.844	3.23	10.43	0.69	0.31	0.747
	50–59	1383	5.60	0.75	0.76	0.21	0.848	3.00	9.00	0.65	0.38	0.666
	60–69	800	6.72	0.82	0.65	0.14	0.831	2.79	7.78	0.55	0.32	0.647
	≥70	327	7.08	0.84	0.70	0.22	0.816	1.87	3.50	0.75	0.65	0.548
Men	18–29	178	5.79	0.76	0.71	0.17	0.821	3.08	9.49	0.83	0.33	0.830
	30–39	461	6.34	0.80	0.70	0.29	0.770	3.04	9.24	0.70	0.25	0.757
	40–49	771	6.83	0.83	0.72	0.19	0.836	3.47	12.04	0.37	0.11	0.651
	50–59	627	7.4	0.86	0.74	0.26	0.816	3.13	9.80	0.33	0.12	0.618
	60–69	402	8.19	0.90	0.69	0.15	0.829	2.57	6.60	0.32	0.18	0.528
	≥70	134	8.10	0.90	0.74	0.40	0.697	2.84	8.07	0.13	0.03	0.487

Abbreviations: VAT = visceral adipose tissue; SAT = subcutaneous adipose tissue; SN = sensitivity; SP = specificity; AUC = area under curve.

## References

[1] Bedogni G., Nobili V., Tiribelli C. (2014). Epidemiology of fatty liver: An update. World J. Gastroenterol..

[2] Volzke H. (2012). Multicausality in fatty liver disease: Is there a rationale to distinguish between alcoholic and non-alcoholic origin?. World J. Gastroenterol..

[3] Bedogni G., Bellentani S., Miglioli L., Masutti F., Passalacqua M., Castiglione A., Tiribelli C. (2006). The Fatty Liver Index: A simple and accurate predictor of hepatic steatosis in the general population. BMC Gastroenterol..

[4] Zelber-Sagi S., Webb M., Assy N., Blendis L., Yeshua H., Leshno M., Ratziu V., Halpern Z., Oren R., Santo E. (2013). Comparison of fatty liver index with noninvasive methods for steatosis detection and quantification. World J. Gastroenterol..

[5] Koehler E.M., Schouten J.N., Hansen B.E., Hofman A., Stricker B.H., Janssen H.L. (2013). External validation of the fatty liver index for identifying nonalcoholic fatty liver disease in a population-based study. Clin. Gastroenterol. Hepatol..

[6] Foschi F.G., Bedogni G., Domenicali M., Giacomoni P., Dall’Aglio A.C., Dazzani F., Lanzi A., Conti F., Savini S., Saini G. (2018). Prevalence of and risk factors for fatty liver in the general population of Northern Italy: The Bagnacavallo Study. BMC Gastroenterol..

[7] Pan J.J., Fallon M.B. (2014). Gender and racial differences in nonalcoholic fatty liver disease. World J. Hepatol..

[8] Tota-Maharaj R., Blaha M.J., Zeb I., Katz R., Blankstein R., Blumenthal R.S., Budoff M.J., Nasir K. (2014). Ethnic and sex differences in fatty liver on cardiac computed tomography: The multi-ethnic study of atherosclerosis. Mayo Clin. Proc..

[9] Lonardo A., Nascimbeni F., Ballestri S., Fairweather D., Win S., Than T.A., Abdelmalek M.F., Suzuki A. (2019). Sex Differences in NAFLD: State of the Art and Identification of Research Gaps. Hepatology.

[10] Chalasani N., Younossi Z., Lavine J.E., Charlton M., Cusi K., Rinella M., Harrison S.A., Brunt E.M., Sanyal A.J. (2018). The diagnosis and management of nonalcoholic fatty liver disease: Practice guidance from the American Association for the Study of Liver Diseases. Hepatology.

[11] Mann R.E., Smart R.G., Govoni R. (2003). The epidemiology of alcoholic liver disease. Alcohol. Res. Health.

[12] Zelber-Sagi S., Nitzan-Kaluski D., Halpern Z., Oren R. (2006). Prevalence of primary non-alcoholic fatty liver disease in a population-based study and its association with biochemical and anthropometric measures. Liver Int..

[13] Chalasani N., Younossi Z., Lavine J.E., Diehl A.M., Brunt E.M., Cusi K., Charlton M., Sanyal A.J. (2012). The diagnosis and management of non-alcoholic fatty liver disease: Practice Guideline by the American Association for the Study of Liver Diseases, American College of Gastroenterology, and the American Gastroenterological Association. Hepatology.

[14] Hunter G.R., Gower B.A., Kane B.L. (2010). Age Related Shift in Visceral Fat. Int. J. Body Compos. Res..

[15] Wajchenberg B.L. (2000). Subcutaneous and visceral adipose tissue: Their relation to the metabolic syndrome. Endocr. Rev..

[16] Demerath E.W., Sun S.S., Rogers N., Lee M., Reed D., Choh A.C., Couch W., Czerwinski S.A., Chumlea W.C., Siervogel R.M. (2007). Anatomical patterning of visceral adipose tissue: Race, sex, and age variation. Obesity (Silver Spring).

[17] Bertoli S., Leone A., Ponissi V., Bedogni G., Beggio V., Strepparava M.G., Battezzati A. (2016). Prevalence of and risk factors for binge eating behaviour in 6930 adults starting a weight loss or maintenance programme. Public Health Nutr..

[18] Lohman T.G., Roche A.F., Martorell R. (1988). Anthropometric Standardization Reference Manual.

[19] Armellini F., Zamboni M., Rigo L., Todesco T., Bergamo-Andreis I.A., Procacci C., Bosello O. (1990). The contribution of sonography to the measurement of intra-abdominal fat. J. Clin. Ultrasound..

[20] Bertoli S., Leone A., Vignati L., Spadafranca A., Bedogni G., Vanzulli A., Rodeschini E., Battezzati A. (2016). Metabolic correlates of subcutaneous and visceral abdominal fat measured by ultrasonography: A comparison with waist circumference. Nutr. J..

[21] Parker R. (2018). The role of adipose tissue in fatty liver diseases. Liver Res..

[22] Manne V., Handa P., Kowdley K.V. (2018). Pathophysiology of Nonalcoholic Fatty Liver Disease/Nonalcoholic Steatohepatitis. Clin. Liver Dis..

[23] Boden G. (1997). Role of fatty acids in the pathogenesis of insulin resistance and NIDDM. Diabetes.

[24] Kelley D.E., Mokan M., Simoneau J.A., Mandarino L.J. (1993). Interaction between glucose and free fatty acid metabolism in human skeletal muscle. J. Clin. Investig..

[25] Saponaro C., Gaggini M., Carli F., Gastaldelli A. (2015). The Subtle Balance between Lipolysis and Lipogenesis: A Critical Point in Metabolic Homeostasis. Nutrients.

[26] Petta S., Gastaldelli A., Rebelos E., Bugianesi E., Messa P., Miele L., Svegliati-Baroni G., Valenti L., Bonino F. (2016). Pathophysiology of Non Alcoholic Fatty Liver Disease. Int. J. Mol. Sci..

[27] Marra F., Bertolani C. (2009). Adipokines in liver diseases. Hepatology.

[28] Friedman S.L. (2008). Hepatic stellate cells: Protean, multifunctional, and enigmatic cells of the liver. Physiol. Rev..

[29] Angulo P. (2006). NAFLD, obesity, and bariatric surgery. Gastroenterology.

[30] Cao Q., Mak K.M., Ren C., Lieber C.S. (2004). Leptin stimulates tissue inhibitor of metalloproteinase-1 in human hepatic stellate cells: Respective roles of the JAK/STAT and JAK-mediated H2O2-dependant MAPK pathways. J. Biol. Chem..

[31] Lord G.M., Matarese G., Howard J.K., Baker R.J., Bloom S.R., Lechler R.I. (1998). Leptin modulates the T-cell immune response and reverses starvation-induced immunosuppression. Nature.

[32] Arner P. (1995). Differences in lipolysis between human subcutaneous and omental adipose tissues. Ann. Med..

[33] Shulman G.I. (2014). Ectopic fat in insulin resistance, dyslipidemia, and cardiometabolic disease. N. Engl. J. Med..

[34] Sam S. (2018). Differential effect of subcutaneous abdominal and visceral adipose tissue on cardiometabolic risk. Horm. Mol. Biol. Clin. Investig..

[35] Fan J.-G., Farrell G.C. (2008). VAT fat is bad for the liver, SAT fat is not!. J. Gastroenterol. Hepatol..

[36] Chang E., Varghese M., Singer K. (2018). Gender and Sex Differences in Adipose Tissue. Curr. Diab. Rep..

[37] Karastergiou K., Smith S.R., Greenberg A.S., Fried S.K. (2012). Sex differences in human adipose tissues—the biology of pear shape. Biol. Sex. Differ..

[38] White U.A., Tchoukalova Y.D. (2014). Sex dimorphism and depot differences in adipose tissue function. Biochim. Biophys. Acta.

[39] Yim J.E., Heshka S., Albu J.B., Heymsfield S., Gallagher D. (2008). Femoral-gluteal subcutaneous and intermuscular adipose tissues have independent and opposing relationships with CVD risk. J. Appl. Physiol..

[40] Schreiner P.J., Terry J.G., Evans G.W., Hinson W.H., Crouse J.R., Heiss G. (1996). Sex-specific associations of magnetic resonance imaging-derived intra-abdominal and subcutaneous fat areas with conventional anthropometric indices. The Atherosclerosis Risk in Communities Study. Am. J. Epidemiol..

[41] Camhi S.M., Bray G.A., Bouchard C., Greenway F.L., Johnson W.D., Newton R.L., Ravussin E., Ryan D.H., Smith S.R., Katzmarzyk P.T. (2011). The relationship of waist circumference and BMI to visceral, subcutaneous, and total body fat: Sex and race differences. Obesity (Silver Spring).

[42] Geer E.B., Shen W. (2009). Gender differences in insulin resistance, body composition, and energy balance. Gend. Med..

[43] Stevens J., Katz E.G., Huxley R.R. (2010). Associations between gender, age and waist circumference. Eur. J. Clin. Nutr..

[44] Meigs J.B., Wilson P.W., Nathan D.M., D’Agostino R.B., Williams K., Haffner S.M. (2003). Prevalence and characteristics of the metabolic syndrome in the San Antonio Heart and Framingham Offspring Studies. Diabetes.

[45] Jensen M.D. (2008). Role of body fat distribution and the metabolic complications of obesity. J. Clin. Endocrinol. Metab..

[46] Seitz H.K., Bataller R., Cortez-Pinto H., Gao B., Gual A., Lackner C., Mathurin P., Mueller S., Szabo G., Tsukamoto H. (2018). Alcoholic liver disease. Nat. Rev. Dis. Primers..

[47] Klop B., do Rego A.T., Cabezas M.C. (2013). Alcohol and plasma triglycerides. Curr. Opin. Lipidol..

[48] Kessenich C.R., Cronin K. (2012). GGT and alcohol consumption. Nurse. Pr..

[49] Bedogni G., Kahn H.S., Bellentani S., Tiribelli C. (2010). A simple index of lipid overaccumulation is a good marker of liver steatosis. BMC Gastroenterol..

[50] Erol A., Karpyak V.M. (2015). Sex and gender-related differences in alcohol use and its consequences: Contemporary knowledge and future research considerations. Drug Alcohol. Depend..

[51] Walker G.E., Marzullo P., Ricotti R., Bona G., Prodam F. (2014). The pathophysiology of abdominal adipose tissue depots in health and disease. Horm. Mol. Biol. Clin. Investig..

[52] Kuk J.L., Saunders T.J., Davidson L.E., Ross R. (2009). Age-related changes in total and regional fat distribution. Ageing Res. Rev..

[53] Ryan A.S., Nicklas B.J. (1999). Age-related changes in fat deposition in mid-thigh muscle in women: Relationships with metabolic cardiovascular disease risk factors. Int. J. Obes. Relat. Metab. Disord..

[54] Kelley D.E., Goodpaster B., Wing R.R., Simoneau J.A. (1999). Skeletal muscle fatty acid metabolism in association with insulin resistance, obesity, and weight loss. Am. J. Physiol..

[55] Bertoli S., Leone A., Vignati L., Bedogni G., Martinez-Gonzalez M.A., Bes-Rastrollo M., Spadafranca A., Vanzulli A., Battezzati A. (2015). Adherence to the Mediterranean diet is inversely associated with visceral abdominal tissue in Caucasian subjects. Clin. Nutr..

[56] Yasutake K., Kohjima M., Kotoh K., Nakashima M., Nakamuta M., Enjoji M. (2014). Dietary habits and behaviors associated with nonalcoholic fatty liver disease. World J. Gastroenterol..

[57] Leone A., Battezzati A., De Amicis R., De Carlo G., Bertoli S. (2017). Trends of Adherence to the Mediterranean Dietary Pattern in Northern Italy from 2010 to 2016. Nutrients.

